# Guava (*Psidium guajava*) Fruit Extract Prepared by Supercritical CO_2_ Extraction Inhibits Intestinal Glucose Resorption in a Double-Blind, Randomized Clinical Study

**DOI:** 10.3390/nu11071512

**Published:** 2019-07-03

**Authors:** Alice König, Bettina Schwarzinger, Verena Stadlbauer, Peter Lanzerstorfer, Marcus Iken, Clemens Schwarzinger, Peter Kolb, Stephan Schwarzinger, Katharina Mörwald, Susanne Brunner, Otmar Höglinger, Daniel Weghuber, Julian Weghuber

**Affiliations:** 1 University of Applied Sciences Upper Austria, 4600 Wels, Austria; 2 Austrian Competence Center for Feed and Food Quality, Safety and Innovation, 4600 Wels, Austria; 3 Johannes Kepler University, Institute for Chemical Technology of Organic Materials, 4040 Linz, Austria; 4 PM International AG, 5445 Schengen, Luxembourg; 5 NBNC—North Bavarian NMR Centre, University of Bayreuth, 95440 Bayreuth, Germany; 6 Obesity Research Unit, Paracelsus Medical University, 5020 Salzburg, Austria; 7 Department of Pediatrics, Paracelsus Medical University, 5020 Salzburg, Austria

**Keywords:** guava extract, oral glucose tolerance test, type 2 diabetes mellitus, supercritical CO_2_ extraction

## Abstract

Inhibition of intestinal glucose resorption can serve as an effective strategy for the prevention of an increase in blood glucose levels. We have recently shown that various extracts prepared from guava (*Psidium guajava*) inhibit sodium-dependent glucose cotransporter 1 (SGLT1)- and glucose transporter 2 (GLUT2)-mediated glucose transport in vitro (Caco-2 cells) and in vivo (C57BL/6N mice). However, the efficacy in humans remains to be confirmed. For this purpose, we conducted a parallelized, randomized clinical study with young healthy adults. Thirty-one volunteers performed an oral glucose tolerance test (OGTT) in which the control group received a glucose solution and the intervention group received a glucose solution containing a guava fruit extract prepared by supercritical CO_2_ extraction. The exact same extract was used for our previous in vitro and in vivo experiments. Blood samples were collected prior to and up to two hours after glucose consumption to quantitate blood glucose and insulin levels. Our results show that, in comparison to the control group, consumption of guava fruit extract resulted in a significantly reduced increase in postprandial glucose response over the basal fasting plasma glucose levels after 30 min (Δ control 2.60 ± 1.09 mmol/L versus Δ intervention 1.96 ± 0.96 mmol/L; *p* = 0.039) and 90 min (Δ control 0.44 ± 0.74 mmol/L versus Δ intervention −0.18 ± 0.88 mmol/L; *p* = 0.023). In addition, we observed a slightly reduced, but non-significant insulin secretion (Δ control 353.82 ± 183.31 pmol/L versus Δ intervention 288.43 ± 126.19 pmol/L, *p* = 0.302). Interestingly, storage time and repeated freeze-thawing operations appeared to negatively influence the efficacy of the applied extract. Several analytical methods (HPLC-MS, GC-MS, and NMR) were applied to identify putative bioactive compounds in the CO_2_ extract used. We could assign several substances at relevant concentrations including kojic acid (0.33 mg/mL) and 5-hydroxymethylfurfural (2.76 mg/mL). Taken together, this clinical trial and previous in vitro and in vivo experiments confirm the efficacy of our guava fruit extract in inhibiting intestinal glucose resorption, possibly in combination with reduced insulin secretion. Based on these findings, the development of food supplements or functional foods containing this extract appears promising for patients with diabetes and for the prevention of insulin resistance. Trial registration: 415-E/2319/15-2018 (Ethics Commissions of Salzburg).

## 1. Introduction

Diabetes mellitus, a chronic disease, is a major public health problem and an independent risk factor for death from heart-related causes [1]. According to the Austrian Diabetes Report, 2017, approximately 415 million people worldwide suffer from diabetes mellitus. By the year 2040, the number of patients will rise to 642 million [1]. Most patients have type 2 diabetes mellitus (T2DM) characterized by a combination of hyperinsulinemia and insulin resistance. The development of T2DM is linked to a more sedentary lifestyle, an energy-rich diet, and obesity. One typical feature of diabetes mellitus is a chronically elevated blood glucose level (hyperglycemia), which can lead to micro- and macrovascular complications such as neuropathy, atherosclerosis, rheumatoid arthritis, retinopathy, end stage renal diseases, and neurodegenerative disorders [2].

Regulating postprandial hyperglycemia provides an important measure in the prevention and management of T2DM. Lower postprandial glucose levels not only reduce the risk of arterial and microvascular diseases but also may have a protective effect on β-cell function [3]. A possible strategy to reduce postprandial glucose and subsequent insulin peaks is the inhibition of intestinal glucose absorption. In general, glucose is absorbed across the small intestine through brush border cells via sodium-dependent glucose cotransporter 1 (SGLT1) and glucose transporter 2 (GLUT2) [4]. The search for appropriate antidiabetic agents has recently been focused on plants, as plant-derived extracts contain bioactive ingredients that inhibit sugar absorption [5]. Some of these phytochemicals, such as polyphenols, have been reported to interact with SGLT1 and GLUT2, resulting in a reduced postprandial glucose response [6]. Moreover, previous studies have identified flavonoids as enhancers of insulin secretion and sensitivity [7]. The antidiabetic effects of different plant- and fruit-derived polyphenols are supported by several studies [8,9,10]. Overall, these studies indicate that some natural extracts might be used as complementary or alternative therapeutic agents to manage, prevent, and/or treat diabetes. However, the efficacy of an extract depends on its bioactive ingredients and, thus, on its raw materials and production method. Before an extract can be used for food products, its efficacy must be confirmed. For instance, a meta-analysis for all available randomized controlled trials comparing the effect of green tea or green tea extract on insulin sensitivity and glycemic control could not confirm the efficacy of green tea in regulating postprandial hyperglycemia. It was found that the consumption of green tea did not decrease the levels of fasting plasma glucose, fasting serum insulin, and oral glucose tolerance test (OGTT)-2 h glucose [11].

Guava (*Psidium guajava*), a tropical fruit, has been used for the treatment of diabetes and other chronic diseases in traditional Chinese medicine for a long time [12]. Some in vitro and in vivo studies have illustrated the antihyperglycemic and hypoglycemic effects of guava leaf extracts [13,14,15]. It seems reasonable that the described effects result from the bioactive compounds of guava. Phytochemical analysis of different parts of guava (peel, flesh, and seed) have demonstrated a high content of total phenolics and flavonoids, including flavanols, flavonols, tannins, and phenolic acid derivatives [16]. We have recently shown that various extracts prepared from guava work as potent SGLT1 and GLUT2 inhibitors in vitro (Caco-2 cells) and in vivo (C57BL/6N mice) [17]. The greatest inhibitory effect was detected for guava fruit extract prepared by supercritical CO_2_ extraction (SFE). Obviously, this extraction procedure results in the isolation of compounds related to the inhibitory effect on glucose resorption at higher concentrations than other techniques such alcoholic extraction.

Based on our previous work, we investigated whether application of this guava extract also leads to a reduced postprandial glucose response in humans. Therefore, we conducted a parallelized, randomized, and placebo-controlled clinical study to confirm the efficacy of guava fruit extract in young healthy adults. In addition, we tested the dependency of storage time on the efficacy of the extract by splitting the intervention group into a September and a November intervention. The obtained results confirmed our hypothesis that the consumption of guava fruit extract together with a glucose solution reduces the extent of the postprandial glucose response during an oral glucose tolerance test (OGTT).

## 2. Materials and Methods

### 2.1. Guava Fruit Extract

The guava fruit extract was prepared by supercritical CO_2_ extraction as previously described [17]. After preparation in August 2018, it was stored at −20 °C until the study was conducted in September 2018. Additionally, the influence of storage stability on the efficacy of the extract was tested in November 2018 with the same batch of guava fruit extract. However, it was frozen and thawed several times before its use.

### 2.2. HPLC-MS, High Pressure Liquid Chromatography-High Resolution Mass Spectrometry

The analysis of the extract was performed using a Thermo Scientific Surveyor HPLC system coupled to a Linear Trap Quadropole (LTQ) Orbitrap Velos. Analyte separation was performed on an Accucore C18 column (150 mm × 3.0 mm inner diameter, 2.6 µm particle size; Thermo Scientific). The column temperature was set to 40 °C, and the injection volume was 1 µL. UV wavelengths were detected at 260 and 360 nm. Analytes were separated by gradient elution, with mobile phase A containing 0.1% formic acid (FA) in water, and mobile phase B containing 0.1% FA in acetonitrile, at a flow rate of 0.5 mL/min. The elution gradient starting conditions were 95% A and 5% B. After 5 min of equilibration time, the proportion of B was increased to 20% at 8 min, to 40% at 12 min, to 60% at 15 min, and to 80% at 17 min. The proportion of B was reduced to 5% from 20 to 25 min. Mass spectra were recorded with an atmospheric pressure chemical ionizer (APCI) in FT mode with a resolution of 60,000. MS2 spectra of the most intense ions were automatically recorded on the LTQ ion trap with collision-induced dissociation utilizing a collision energy of 35%.

### 2.3. GC-MS, Gas Chromatography-Mass Spectrometry

The extract was analyzed on a Thermo Scientific Trace GC equipped with a capillary column Thermo TRX35MS (30 m × 0.32 mm × 0.25 µm) and a Thermo Scientific Polaris ion trap mass spectrometer. The GC column temperature conditions were as follows: Initial temperature 40 °C, hold for 2 min, increase at 16 °C min^−1^ to 300 °C, and hold this temperature for 5 min. A programmed temperature vaporizer (PTV) injector was used at 250 °C with helium gas flow of 2.5 mL min^−1^ and a split ratio of 1:25. Mass spectra were recorded under electron impact ionization at 70 eV electron energy in the range of *m*/*z* 15–650.

### 2.4. NMR, Nuclear Magnetic Resonance Spectroscopy

Pre-saturated 1D-^1^H NMR spectra of the SFE-extract were obtained on a 600 MHz Bruker Avance II NMR Spectrometer equipped with a z-axis HCN-triple resonance probe at 298 K. The sample was prepared according to the protocol for fruit juice profiling [18]. Following two dummy scans, a total of 32 transients was acquired with 32k data points, a spectral width of 16 ppm, and a receiver gain of 14. For optimal water suppression by pre-saturation the center frequency was placed at the position of the water signal at 4.7 ppm. Spectra were processed with an exponential multiplication with a line broadening of 0.3, Fourier-transformed and phase-corrected. Major compounds were assigned by spectral comparison with in-house databases, the Human Metabolome Database [19], and the Biological Magnetic Resonance Data Bank Metabolomics [20], respectively.

### 2.5. Study Design

The study was performed in a randomized, double-blind, parallelized design. It was conducted in accordance with the Declaration of Helsinki, and the protocol was approved by the Ethics Committee of Salzburg (415-E/2319/15-2018). The sample size was estimated following a recent EMA guideline (European Medicines Agency 2015) [21], calculations were based on the formula for a two-sided test in an analysis of covariance model with the basal fasting plasma glucose concentration at baseline as the covariate. Additionally, the Guenter–Schouten adjustment was applied, in order to account for the fact that a rather small sample size was expected [22]. Healthy subjects of normal weight (i.e., BMI < 25) were recruited from the Paracelsus Medical University Salzburg. All study participants gave their informed consent for inclusion before they participated in the study. The exclusion criteria for selecting the subjects were as follows: Diabetes mellitus (type 1 and 2), pregnancy or lactation, indigestion-causing diseases (including fructose malabsorption), acute infectious disease, and known allergies to the contents of the glucose solution in use. In total, 32 volunteers aged between 19 and 29 were approved for the study, one of whom canceled due to an allergic reaction on the day before the study. Thus, 31 subjects (20 female and 11 male) were randomized using an internet-based tool (QuickCalcs, GraphPad, San Diego, California) into a control (*n* = 15) or intervention group (*n* = 16). The trial was performed on three different days in order to test if the efficacy and storage stability of the same guava fruit extract is controlled by its time of storage. The first two experiments were conducted on the 5th and 12th of September 2018 to confirm the efficacy of the guava fruit extract in young healthy adults and were combined as the ‘September intervention’. The third experiment was executed on the 28th of November to investigate the extract’s efficacy after prolonged storage time. The CONSORT diagram in Supplementary Figure S1 presents an overview of the enrollment of the subjects, their allocation to treatment, and how they were analyzed in the trial. Table 1 summarizes the relevant data of the subjects in the control and intervention groups. Volunteers were requested to avoid excessive physical exercise and smoking prior to the study. They were requested not to eat or drink anything except water for ten hours before measuring fasting plasma blood glucose levels. Furthermore, all subjects recorded details of their medication, sleeping duration, and last meal on the previous day. The self-reports can be found in Supplementary Table S1. After an overnight fast, each participant performed an oral glucose tolerance test (OGTT), in which the members of the control group each received a glucose solution (75 g glucose) and the members of the intervention group each received a glucose solution also containing 2.5 mL of the guava fruit extract. Each glucose solution was prepared by dissolving one package of Glucoral 75 Citron (Germania, Pharmazeutika GesmbH, Vienna, Austria) in 300 mL of water. The glucose solutions with fruit extract were served such that they could not be distinguished from those without fruit extract by appearance or flavor. So, neither the study director nor the subjects knew who received which treatment. Subjects consumed the solutions within 5 min. Blood samples were collected prior to and 15, 30, 60, 90, and 120 min after glucose consumption. The samples were processed immediately to quantitate plasma blood glucose and insulin levels.

### 2.6. Sample Preparation

Blood samples were drawn using a 21G Safety Blood Collection Set (Greiner Bio-One GmbH, Kremsmünster, Austria) in combination with Vacuette tubes for Li-Heparin-plasma (Greiner Bio-One GmbH, Kremsmünster, Austria) and handled according to preanalytical guidelines [23]. Plasma was prepared by centrifugation of the blood samples at 1500 × g for 10 min at 22 °C.

### 2.7. Plasma Blood Glucose and Insulin Determination

Plasma blood glucose and insulin were measured using standard techniques at the central laboratory of the University Hospital Salzburg. Enzymatic determination-absorption photometry was carried out for the quantitative determination of glucose in plasma using an automated cobas^®^ 8000 analyzer with a c702 module (Roche, Mannheim, Germany). Plasma insulin concentrations were measured by a chemiluminescent microparticle immunoassay (CMIA) using an Architect Insulin assay (Abbott, Chicago, IL, USA).

### 2.8. Calculations and Statistics

The areas under the curve (AUCs) for glucose and insulin were calculated for each experimental condition as the area beneath the curve and above the fasting level from 0 to 120 min using GraphPad Prism 6.1 for Windows (GraphPad Software Inc., San Diego, CA, USA). Screening for normality was done by the Shapiro–Wilk test. If the results were normally distributed, the t-test with Welch’s correction was used, otherwise we used the Mann–Whitney test. Significance testing was performed with GraphPad Prism 6.1 for Windows. Differences were considered significant with *p* < 0.05 for unpaired t-tests with Welch’s correction. All values are presented as the means ± SD, if not otherwise stated.

## 3. Results

### 3.1. Influence of Guava Fruit Extract on Postprandial Plasma Glucose Response

**Figure 1:**
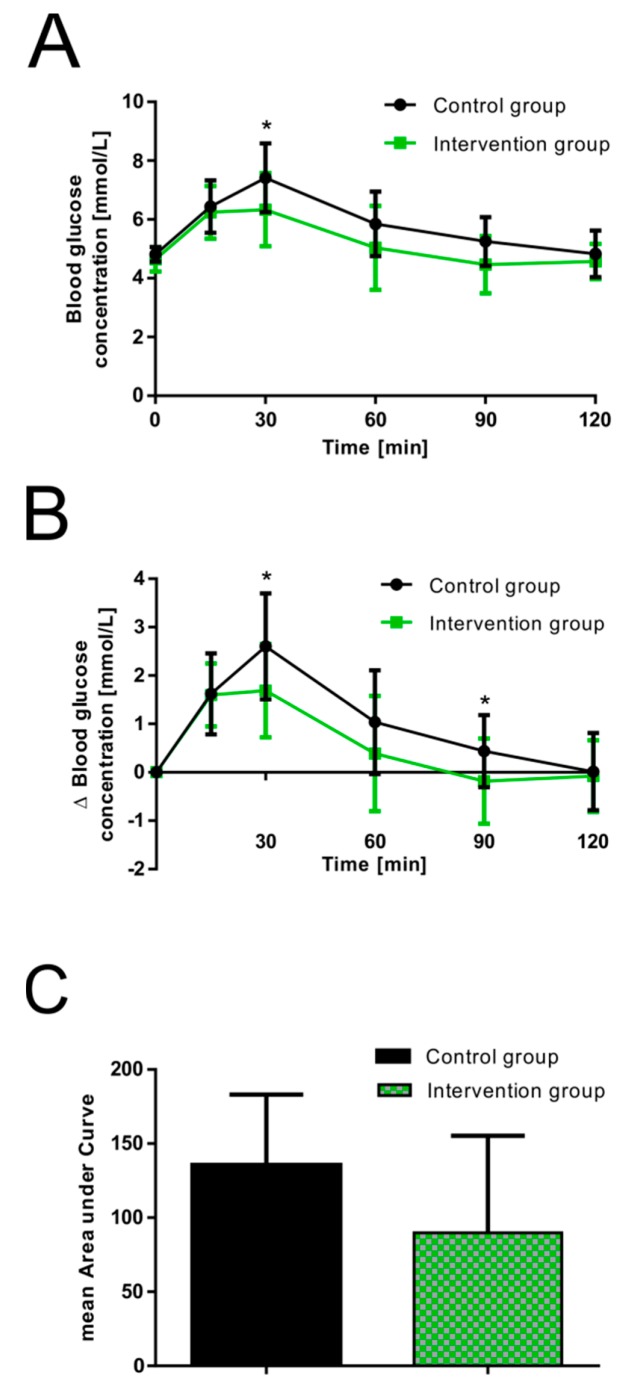
Effect of 2.5 mL guava fruit extract on postprandial glucose response [mmol/L] in healthy male and female volunteers. (**A**) Absolute changes in mean postprandial glucose responses over 120 min compared to control. (**B**) Relative changes in the mean postprandial glucose response over the basal fasting plasma glucose levels over 120 min compared to the control. (**C**) Calculated mean areas under the curve based on relative changes in glucose responses. Values are presented as the means ± SD; control group: *n* = 15 and intervention group: *n* = 10. * *p* < 0.05.

The basal fasting plasma glucose concentration of the subjects in the control and the September intervention group (combined results of studies performed on the 5th and 12th of September) was examined before consumption of the glucose solutions, which resulted in a mean value of 4.75 ± 0.33 mmol/L (control 4.81 ± 0.25 mmol/L versus September intervention 4.65 ± 0.42 mmol/L, *p* = 0.275). None of the participants in either study group showed hypoglycemia at baseline. Plasma glucose levels reached peak values 30 min after oral glucose consumption in both groups (absolute glucose concentrations, Figure 1A), gradually declined over the remaining test period, and reached basal fasting values after two hours. A significantly decreased peak blood glucose level was detected for guava fruit extract at 30 min (6.33 ± 1.25 mmol/L, *p* = 0.043) when compared to the control group (7.41 ± 1.17 mmol/L). Reduced mean plasma glucose concentrations were also found for the September intervention group at 60 and 90 min. The lowest observed postprandial glucose concentration with a value of 3.39 mmol/L was found for one participant in the September intervention group at time points 60 and 90 min. However, the glucose level increased and a concentration of 4.72 mmol/L was measured at time point 120 min. The absolute change in blood glucose concentrations over baseline levels is presented as the increase in postprandial blood glucose response over the basal fasting plasma glucose levels (relative change (Δ), Figure 1B). The September intervention group showed a significantly decreased peak blood glucose response (Δ) after 30 min (1.69 ± 0.96 mmol/L, *p* = 0.039) compared to the control (2.60 ± 1.09 mmol/L). No significant differences were found between the groups at 60 and 90 min. However, a reduced increase in postprandial glucose response was observed for the September intervention group also at these time points.

Based on the relative change in postprandial glucose response, the mean area under the curve (AUC) was calculated for both groups. As illustrated in Figure 1C, a reduced (close to significance) AUC was found for the September intervention group (135.5 ± 47.5 mmol/L*min for control, 89.7 ± 65.5 mmol/L*min for the September intervention group, *p* = 0.076).

### 3.2. Influence of Guava Fruit Extract on Postprandial Plasma Insulin Response

**Figure 2:**
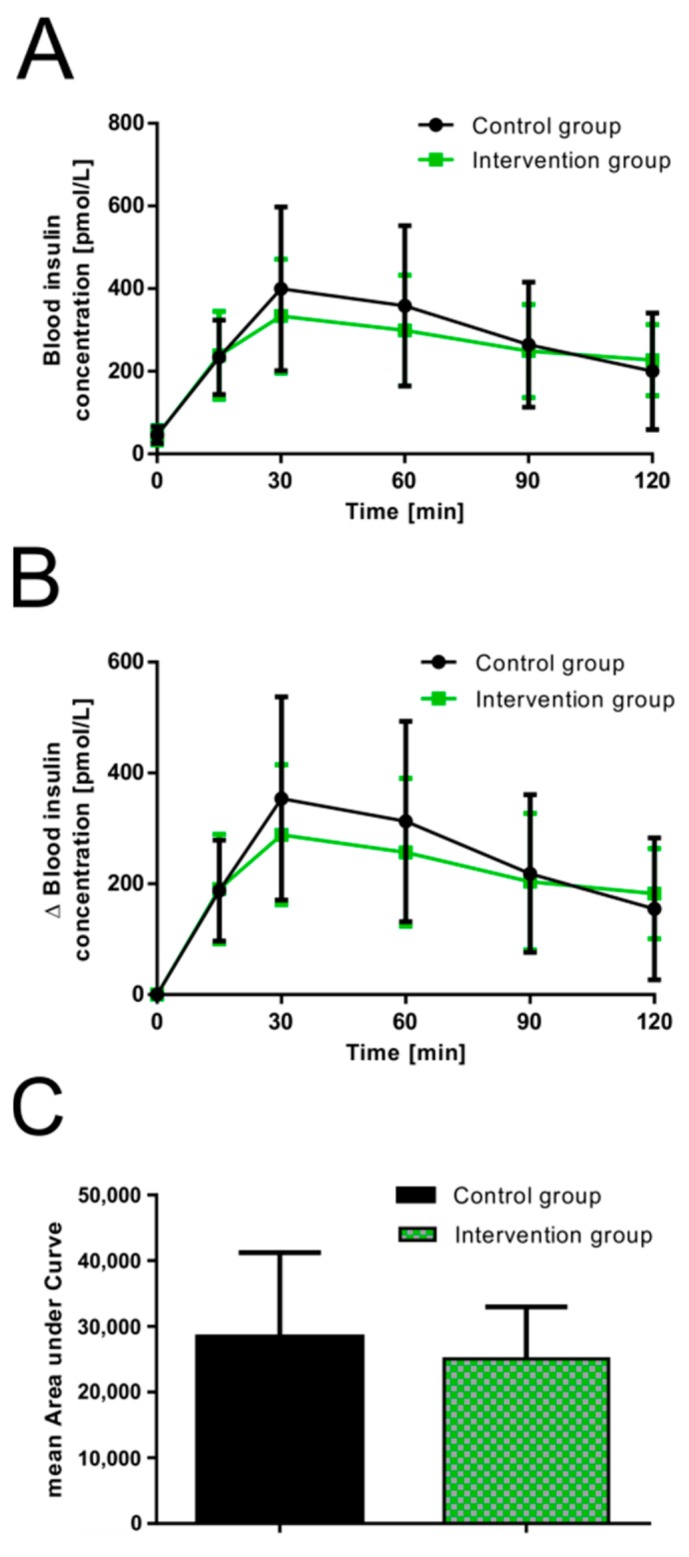
Effect of 2.5 mL guava fruit extract on postprandial insulin secretion [pmol/L] in healthy male and female volunteers. (**A**) Absolute changes in mean postprandial insulin responses over 120 min compared to control. (**B**) Relative changes in mean postprandial insulin concentrations over the basal fasting plasma insulin levels over 120 min compared to control. (**C**) Calculated mean areas under the curve based on relative changes in insulin responses. Values are presented as the means ± SD; control group: *n* = 15 and intervention group: *n* = 10.

The averaged plasma insulin concentration of the overnight fasting samples was 45.56 ± 21.13 pmol/L (control 45.98 ± 20.53 pmol/L versus September intervention 44.93 ± 23.12 pmol/L, *p* = 0.989) and increased as expected after consumption of the glucose solutions. Peak plasma insulin levels were reached at 30 min in the control (absolute change: 399.80 ± 198.15 pmol/L; relative change: 353.82 ± 183.31 pmol/L) and September intervention groups (absolute change: 333.36 ± 137.50 pmol/L; relative change: 288.43 ± 126.19 pmol/L). As indicated in Figure 2A (absolute insulin concentrations) and Figure 2B (relative change in insulin concentrations (Δ)), the presence of guava fruit extract resulted in a trend of reduced postprandial insulin secretion at 30 and 60 min. Accordingly, the mean AUC of insulin was also lower for the September intervention group (Figure 2C; control 28.4 ± 12.9 nmol/L*min versus September intervention 25.1 ± 7.9 nmol/L*min, *p* = 0.435).

### 3.3. The Efficacy of Guava Fruit Extract Depends on Its Storage Time

We tested the efficacy and storage stability of the guava fruit extract by comparing the results from the study conducted in September 2018 (described above) with those obtained from the trial in November 2018. For the last-mentioned study, the same batch of guava fruit extract was used; however, it was frozen and thawed several times before its use.

**Figure 3:**
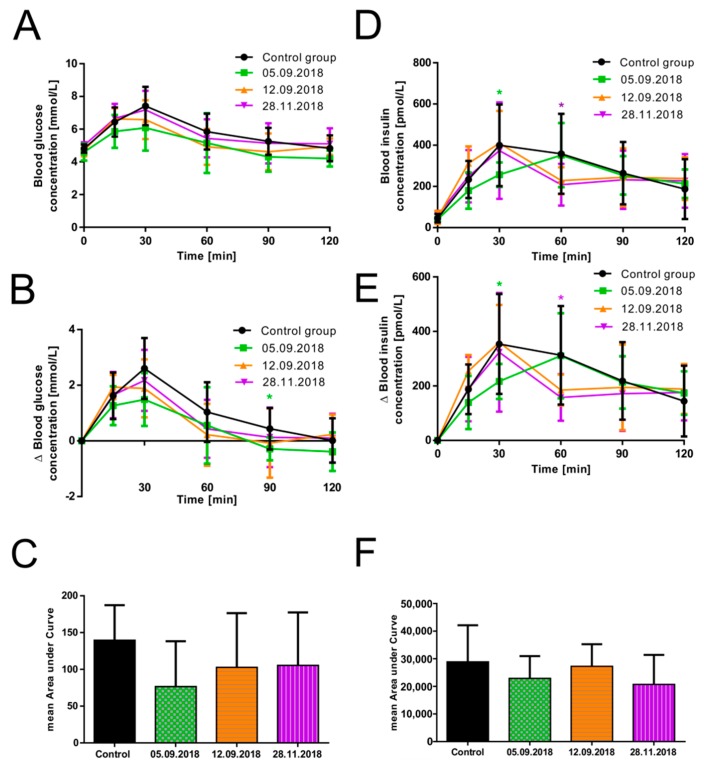
Impact of the storage time of guava fruit extract on its efficacy regarding (**A**) absolute changes in mean postprandial glucose concentration [mmol/L] over 120 min, (**B**) relative changes in mean postprandial glucose concentration [mmol/L], (**C**) calculated mean areas under curve based on relative changes in glucose responses, (**D**) absolute changes in mean postprandial insulin responses [pmol/L] over 120 min, (**E**) relative changes in mean postprandial insulin responses [pmol/L], and (**F**) calculated mean areas under curve based on relative changes in insulin responses. Values are presented as the means ± SD; control group: *n* = 15, 5th September 2018: n = 5, 12th September 2018: *n* = 5, and 28th November 2018: *n* = 6. * *p* < 0.05, significantly different from control.

As described before, the volunteers were divided into a control group (*n* = 15, all subjects who received the glucose solution without guava fruit extract) and an intervention group (*n* = 16, all subjects who received the glucose solution with guava fruit extract). The intervention group itself was split into three smaller groups. On the two study days performed on the 5th of September 2018 and the 12th of September 2018, the intervention group consisted of five subjects each. For the third trial performed on the 28th of November 2018, subjects performed the same oral glucose tolerance test (November intervention group *n* = 6), and changes in plasma blood glucose and insulin concentrations were monitored. As indicated in Figure 3A, B, the presence of the guava fruit extract resulted in a reduced increase of the mean postprandial blood glucose concentration on all three trial days. However, the efficacy of the extract seemed to decrease with prolonged storage time: The strongest effect was observed on the 5th of September 2018, with a significant difference after 90 min for the relative change in glucose concentration (*p* = 0.017) and an obvious effect by trend at 30 and 60 min. The efficacy of the guava extract was slightly lower for the trial performed on the 12th of September and especially lower for the study conducted on the 28th of November. The largest mean AUC was calculated for the control group (139.6 ± 47.7 mmol/L*min) and the smallest mean AUC for the intervention group participating on the 5th of September (76.7 ± 61.7 nmol/L*min, *p* = 0.085; Figure 3C). Similar mean AUC values were calculated for the two other study days (102.8 ± 73.6 mmol/L*min, *p* = 0.341 and 105.5 ± 71.9 mmol/L*min, *p* = 0.320).

We also quantified the postprandial insulin secretion at various time points for all three trial days. As shown in Figure 3D–F, we detected a significantly decreased mean postprandial insulin secretion (216.69 ± 64.25 pmol/L, *p* = 0.024; relative change) at the 30 min time point for the study performed on the 5th of September 2018. The mean peak plasma insulin level was first reached after 60 min and decreased thereafter. Insulin secretion for the studies performed on the 12th of September (184.97 ± 56.71 pmol/L, *p* = 0.177; relative change at 60 min) and the 28th of November 2018 (157.7 ± 85.4 pmol/L, *p* = 0.043; relative change) appeared to be similar, with the mean insulin peak at the 30 min time point comparable to the control group but a significant reduction for the study performed on 28th of November 2018 at the 60 min time point. Finally, the mean AUC of insulin was calculated based on the relative change in postprandial insulin responses for each trial day. In comparison to the control group (28.88 ± 13.27 nmol/L*min), the mean AUC for all three trial days showed a decreasing trend, with the smallest values for the studies performed on the 5th of September (22.9 ± 8.1 nmol/L*min, *p* = 0.25) and 28th of November 2018 (20.7 ± 10.7 nmol/L*min, *n* = 0.1684).

Taken together, storage time and freeze-thawing repetitions negatively influenced the efficacy of the guava extract.

### 3.4. HPLC-MS, GC-MS, and NMR Analysis of Guava Fruit Extract

In our previous in vitro and in vivo study describing the inhibitory effect of various guava extracts on intestinal glucose resorption, we used HPLC analysis to detect and quantitate bioactive compounds based on available standards and known retention times [17]. For a better characterization of the guava extract prepared by SFE used for this study, we applied HPLC-MS, GC-MS, and NMR to identify additional compounds. As shown in the chromatograms in Supplementary Figures S2–S4 and Supplementary Tables S2–S4, several substances could be identified with HPLC-MS, GC-MS, and NMR. 5-Hydroxymethylfurfural (2.76 mg/mL) could be detected with all three methods, whereas kojic acid (0.33 mg/mL) was detected only with HPLC-MS and NMR. The compound is too polar to be detected in gas chromatography. In addition, 2,3-butanediol, acetic acid, and ethanol could be identified by GC-MS and NMR, as well as some other compounds present at very low concentrations.

Compounds usually found in guava extracts prepared by alcoholic or aqueous extraction procedures could not be assigned in significant amounts based on the analytical results obtained.

## 4. Discussion

Previous research has indicated that consumption of different guava fruit extracts reduces postprandial glucose peaks by inhibition of the intestinal glucose transporters SGLT1 and GLUT2 in vitro (Caco-2 cells) and in vivo (C57BL/6N mice) [17]. Here, we determined the potential of guava fruit extract prepared by supercritical CO_2_ extraction to regulate postprandial blood glucose response in healthy young adults during an oral glucose tolerance test (OGTT).

The OGTT is commonly used in clinical research to evaluate glucose tolerance and thus provides an appropriate method to assess postprandial glucose response in humans [24]. As expected, the consumption of 2.5 mL of guava fruit extract reduced postprandial mean blood glucose levels in healthy students, as shown by comparing the results of the OGTT between the intervention and control groups. A significantly decreased peak blood glucose level was detected at 30 and 90 min for the group that consumed guava extract. One of the participants in the September intervention group even showed a postprandial glucose concentration of 3.39 mmol/L, which goes below the threshold of plasma glucose ≤ 3.9 mmol/L for the definition of hypoglycemia, according to the American Diabetes Association/European Medicines Agency specified guidelines [25]. Nevertheless, no other symptoms of hypoglycemia were observed, and the person behaved inconspicuously. Moreover, the glucose concentration increased again afterwards. In fact, glucose concentrations of <3.0 mmol/L can cause cognitive dysfunction and are sufficiently low to indicate serious, clinically important hypoglycemia. A glucose concentration above this value, as it was measured in this study, need not be reported in clinical trials [26]. Blood glucose regulation was accompanied by a modestly reduced, but nonsignificant, postprandial insulin secretion.

The present findings seem to be consistent with other clinical research on plant-derived extracts. For instance, mulberry leaf extract significantly reduced total blood glucose and insulin secretion after ingestion of maltodextrin in a randomized, double-blind, placebo-controlled study. The authors reported a classical dose–response curve with significant effects over placebo [27]. It is interesting to note that the desired effects in our study were achieved at low concentrations of only 2.5 mL (0.036 mL per kg bodyweight; 70 kg total body weight assumption) of the guava fruit extract. This was significantly lower (approximately 10 times) than the applied extract concentration in our previous mouse study (0.4 mL per kg body weight) [17]. It can be assumed that higher concentrations of the extract would lead to a stronger reduction in postprandial glucose response.

It is likely that bioactive compounds are relevant for the observed effects. In this context, polyphenols are known to positively influence postprandial glycemic responses and fasting hyperglycemia, as well as acute insulin secretion and insulin sensitivity [28]. *P. guajava* has been reported to contain a variety of phenolic compounds [16]. For this study, guava fruit extract was prepared by supercritical CO_2_ extraction (SFE). A detailed phytochemical composition of guava fruit extract prepared by SFE still needs to be identified and investigated [17]. To date, SFE has only been applied for guava leaves and seeds [29], whereas this technology has not been used for guava fruit. In general, supercritical fluid extraction is safe, clean, and environmentally friendly. Carbon dioxide, which is widely used, is an inert gas with many technological advantages. It is readily available, cheap, odorless, tasteless, and generally regarded as safe [30]. For that reason, SFE is an attractive technology to obtain bioactive compounds for application in the food industry [31].

Here, we show for the first time that a guava fruit extract prepared by SFE can effectively reduce postprandial glucose peaks in humans. Regulating postprandial hyperglycemia could prevent various complications of T2DM, such as microvascular diseases [3]. For this purpose, bioactive compounds in plant-derived extracts that inhibit sugar absorption are of great interest [8,9,10]. In response to the increasing demand for natural and plant-based foods, some of these extracts could be used as an alternative to oral synthetic hypoglycemic agents [32]. The study thus lays a basis for the development of antidiabetic food products based on guava fruit extract.

Finally, the storage stability of guava fruit extract was investigated. Interestingly, the same batch of extract was less effective after a prolonged storage time and repeated freezing and thawing. After preparation in August 2018, the effect of the guava fruit extract showed a trend toward a reduced blood glucose increase with a significant difference at 90 min on the 5th of September 2018. To our surprise, this trend was less remarkable one week later. Freezing and thawing and storage until November had an adverse influence on the efficacy of the extract, as the glucose response was only slightly reduced on the 28th of November. These findings have important implications for developing food supplements or functional foods, as they indicate that the guava fruit extract is not stable under certain circumstances.

To identify potential bioactive compounds, we analyzed the extract by HPLC-MS, GC-MS, and NMR. Surprisingly, we did not detect expected polyphenolic compounds such as phloretin, catechins, guaijaverin, or quercetins. As HPLC-MS analysis alone resulted in a chromatogram indicating several compounds of a distinct mass at various retention times, which could not be clearly allocated based on existing libraries, we used GC-MS and NMR to address this question. Fortunately, we could identify several compounds, two of them being of special interest: Kojic acid and 5-hydroxymethylfurfural. First, both substances have been reported in the context of antidiabetic properties [33,34]. Second, in HPLC analysis, they represented some of the major peaks and were therefore the compounds present at the highest concentrations. Third, both compounds were also found in ethanolic extracts prepared from guava leaves and fruits that were also effective in our previous in vitro studies [17]. Based on these facts it seems feasible that the described inhibitory effect on intestinal glucose resorption is related to presence of kojic acid and/or 5-hydroxymethylfurfural. As the guava extract prepared by SFE was the most effective one in vitro (Caco-2 cells) and in vivo (mouse) [17], we chose to use it for this trial. In addition, the technology offers several advantages: On the one hand, CO_2_ is cheap, safe, and physiologically sound to very low levels. Therefore it is approved for food products without declaration [35]. On the other hand, CO_2_-based SFE results in the enrichment of other compounds than those enriched by alcoholic extraction procedures. It dissolves non-polar or slightly polar, low molecular weight compounds and oxygenated organic compounds of medium weight, but excludes free fatty acids, pigments, water, proteins, polysaccharides, sugars, and mineral salts [35,36]. Consequently, extracts prepared by SFE contain putative bioactive compounds in a different composition than those prepared by traditional methods, including alcoholic or aqueous extraction techniques.

To our knowledge, this is the first report showing a beneficial effect of guava fruit extract prepared by SFE on glucose response in healthy young adults. This study has confirmed our hypothesis that consumption of guava fruit extract leads to a reduced postprandial glucose response in humans.

Finally, some limitations need to be considered. First, the number of subjects participating in this study was relatively small. Therefore, the findings need to be interpreted cautiously. However, based on the reported effects in vitro and in vivo [17] and the calculated significance (this study), the efficacy of the guava extract prepared by SFE appears to be reliable. Second, an issue that was not addressed in this study was whether there exists a difference between men and women. However, we have shown previously that the used guava extract reduces glucose transport by a specific inhibition of glucose transporter 2 (GLUT2) [17], which is not a gender dependent transport system. Third, as described before, we have used low concentrations of the guava extract for this study. To get a stronger effect one could increase the concentration, but excessive consumption of the guava extract could lead to diarrhea and flatulence. However, relevant symptoms were not observed in our previous mouse study [17]. Finally, for application in antidiabetic food products for day-to-day consumption, long-term effects and potential side effects have yet to be determined. In addition, the efficacy of the extract in patients with T2DM is still unknown, as the study was conducted with young, healthy volunteers. Therefore, further studies are necessary to evaluate the potential of guava fruit extract prepared by SFE for the prevention of T2DM and the regulation of hyperglycemia in affected patients.

## Figures and Tables

**Table 1:** Relevant data of the subjects in the control and intervention groups. Values shown are the mean ± standard deviation (SD). A t-test with Welch’s correction was used to compare height and body weight between the groups. A Mann–Whitney test was used to compare body mass index (BMI) and abdominal girth (marked in green). The chi-square test was used for gender analysis. n = number of subjects.

	Control Group		September Intervention		*p*-Value	November Intervention		*p*-Value
	Mean	± SD	Mean	± SD		Mean	± SD	
Subjects [n]	15		10			6		
Males/Females [n]	6/9		3/7			2/4		
Height [cm]	172.73	7.85	168.74	8.69	0.2582	176.3	8.18	0.3842
Weight [kg]	69.1	11.36	62.52	9.38	0.129	67.93	7.48	0.7875
Body mass index [kg/m^2^]	23.06	2.73	21.88	2.19	0.1936	21.82	0.67	0.36812
Abdominal girth [cm]	78.5	10.49	72.5	6.75	0.1936	75.58	7.31	0.56192

## Supplementary Materials

The following are available online at https://www.mdpi.com/2072-6643/11/7/1512/s1, Figure S1: Guava study CONSORT diagram. Figure S2: HPLC-MS analysis of guava fruit extract prepared by supercritical CO_2_ extraction. A diode array detector (DAD) chromatogram (top) and the total ion current (TIC) chromatogram (bottom) are shown. *Kojic acid (0.33 mg/mL, **5-hydroxymethylfurfural (2.76 mg/mL). Figure S3: GC-MS analysis of guava fruit extract prepared by supercritical CO_2_ extraction. *5-Hydroxymethylfurfural. Figure S4: NMR ^1^H-1D NMR spectrum of guava fruit extract prepared by supercritical CO_2_ extraction. Upper panel shows the low range of the NMR-chemical shift scale (1.0 to 4.7 ppm) relevant for methanol, ethanol, acetic acid, and 2,3-butanediol. Lower panel depicts the region from 4.5 to 9.6 ppm relevant for 5-hydroxymethylfurfural, kojic acid, and formic acid, respectively. The grey bar indicates residual water from water suppression. For better visibility, the signal intensities in both panels have been optimized (visible in the box with the broken line for signals belonging to 5-hydroxymethylfurfural and kojic acid). Table S1: Subject self-reports on medication, meal identity, and sleeping duration. Table S2: HPLC-MS analysis of guava fruit extract prepared by supercritical CO_2_ extraction. Identification of compounds marked with an asterisk is based on exact mass, fragmentation analysis, and comparison with GC-MS results. The mass to charge ratio (*m*/*z*) represents the exact molecular weight of the protonated ion, which was fragmented resulting in the representative ions listed in section MS2. Table S3: GC-MS analysis of guava fruit extract prepared by supercritical CO_2_ extraction. Identification of compounds is based on comparison of the mass spectra to the National Institute of Standards and Technology (NIST) library. Table S4: Assignments of substances identified by NMR spectroscopy.
