# Supplementary material for: Guava (Psidium guajava) Fruit Extract Prepared by Supercritical CO2 Extraction Inhibits Intestinal Glucose Resorption in a Double-Blind, Randomized Clinical Study
Source: Nutrients. 2019 Jul 3;11(7):1512. doi: 10.3390/nu11071512 (PMC6683095; doi:10.3390/nu11071512)
Supplement: Supplementary file 1 [file nutrients-11-01512-s001.pdf]

Supplementary Table S1. Subject self-reports on medication, meal identity and sleeping duration.

**Control**	**Actual medication**	**Last meal the day before**	**Sleep behavior**
1	no	19:00 cake and coffee	01:00 /length 5,5h
2	no	18.00 wrap, cheese, tomatoes	00:20 /length 6h
3	no	16:00 ice cream	22:00 /length 8h
4	no	12:00 bread, cheese, Coca-Cola	23:30 /length 6h
5	Nuva Hormon Ring	21:00 mango, cereal bar, water	23:00 /length 7h
6	Ceracette	21:00 Chilli con Carne	23:30 /length 7h
7	no	21:00 Coca-Cola, crisps, soup	23:00 /length 7h
8	no	19:30 popcorn	22:00 /length 8,5h
9	no	19:00 coffee, banana, rice cakes with peanut butter, chocolate biscuits	23:00 /length 7,5h
10	no	19:00 coffee, banana, rice cakes with peanut butter, chocolate biscuits	23:00 /length 7,5h
11	no	20:30 soft drink Saft, corn tortillas	24:00 /length 6,5h
12	no	21:10 M&Ms	23:00 /length 6,5h
13	no	19:00 crispbread with cheese, tomatoes, cucumber	22:30 /length 7h
14	no	21:30 chicken, broccoli, bread	00:00 /length 7h
15	no	20:00 Spelled pasta, red pesto, lettuce, olives, tomatoes, basil, oil	22:00 /length 8h
**September Intervention**	**Actual medication**	**Last meal the day before**	**Sleep behavior**
16	no	20:00 chicken, potatoes	23:00 /length 7h
17	no	20:00 yogurt, apple	23:00 /length 7h
18	no	21:00 rice, vegetables	23:00 /length 5h
19	coil	21:00 Asian noodles, water	23:30 /length 7h
20	Yris	21:00 Chilli con Carne, Coca-Cola	23:00 /length 7h
21	no	20:30 burger, chips, cider	23:30 /length 7h
22	Monostep	18:00 tomato-avocado salad with baguette	23:00 /length 8h
23	Thyresch 125 mg	20:30 burger, pommes, cider	23:30 /length 7h
24	Topiramat 75 mg; Madonella and Deforatadin (each one tablet)	19:00 pasta, vegetables	23:45 /length 7h
25	no	21:00 gummy bears, sausage roll	24:00 /length 6h
**November Intervention**	**Actual medication**	**Last meal the day before**	**Sleep behavior**

26	no	21:00 sushi	23:00 /length 8,6h
27	no	20:00 lasagna, water	21:00 /length 7h
28	no	20:30 minced meat with sweet potatoes, sprinkled apple spritzer	23:30 /length 7h
29	no	21:00 pasta, soft drink	23:30 /length 9h
30	no	21:00 fruit, herb tea	23:00 /length 6,5h
31	no	22:00 ice cream	23:50 /length 6h

Supplementary Table S2. HPLC-MS analysis of guava fruit extract prepared by supercritical CO_2 extraction. Identification of compounds marked with an asterisk is based on exact mass, fragmentation analysis and comparison with GC-MS results. The mass to charge ratio (m/z) represents the exact molecular weight of the protonated ion, which was fragmented resulting in the representative ions listed in section MS2.

tR /UV	Compound	m/z	MS2
13.22		-	
12.96		-	
12.74		247.1327	229, 219, 201, 187, 163, 137
10.84		263.1276	245, 233, 205, 203, 165
10.10		182.0811 + 136.0757	
8.37		265.1404	248, 235, 229, 217, 203, 193
7.72		194.0789	166, 136
7.07		-	
4.05	3,5-Dihydroxy-2-methyl-4H-pyran-4-one*	143.0338	125, 124, 115, 96
3.91		-	
2.72	Hydroxymethylfurfural	127.0389	108
2.20	2,3-Dihydro-3,5-dihydroxy-6-methyl-4H-pyran-4-one*	145.0494	127, 98
1.66	Kojic acid	143.0338	125, 97, 67
1.36		123.0553	106, 95, 81, 79

Supplementary Table S3. GC-MS analysis of guava fruit extract prepared by supercritical CO_2 extraction. Identification of compounds is based on comparison of the mass spectra to the NIST library.

tR	compound
0,-.88-2	Water, ethanol, and other low molar mass compounds
2.7	Acetic acid
3.95	Propylene glycol
4.30	2,3-Butanediol (isomer 1)
4.46	2,3-Butanediol (isomer 2)
4.96	Furfural
7.58	Pantolactone
8.40	2,3-Dihydro-3,5-dihydroxy-6-methyl-4H-pyran-4-one
8.86	3,5-Dihydroxy-2-methyl-4H-pyran-4-one
9.47	m/z=131, 86, 85, 57, 45
9.62	5-Hydroxymethylfurfural

Supplementary Table S4. Assignments of substances identified by NMR spectroscopy.

**Compound**	**NMR-chemical shift in ppm and multiplicity in brackets**			
Ethanol	1.17 (t)	3.65 (q)	-	-
Acetic acid	2.08 (s)	-	-	-
2,3 Butanediol	1.13 (d)	3.61 (d)	3.71 (d)	
Formic acid	8.26 (s)	-	-	-
Methanol	3.35 (s)	-	-	-
5-Hydroxymethylfurfural	4.69 (s)	6.67 (d)	7.53 (d)	9.44 (s)
Kojic acid	4.52 (s)	6.59 (s)	8.10 (s)	

Supplementary Figure 1

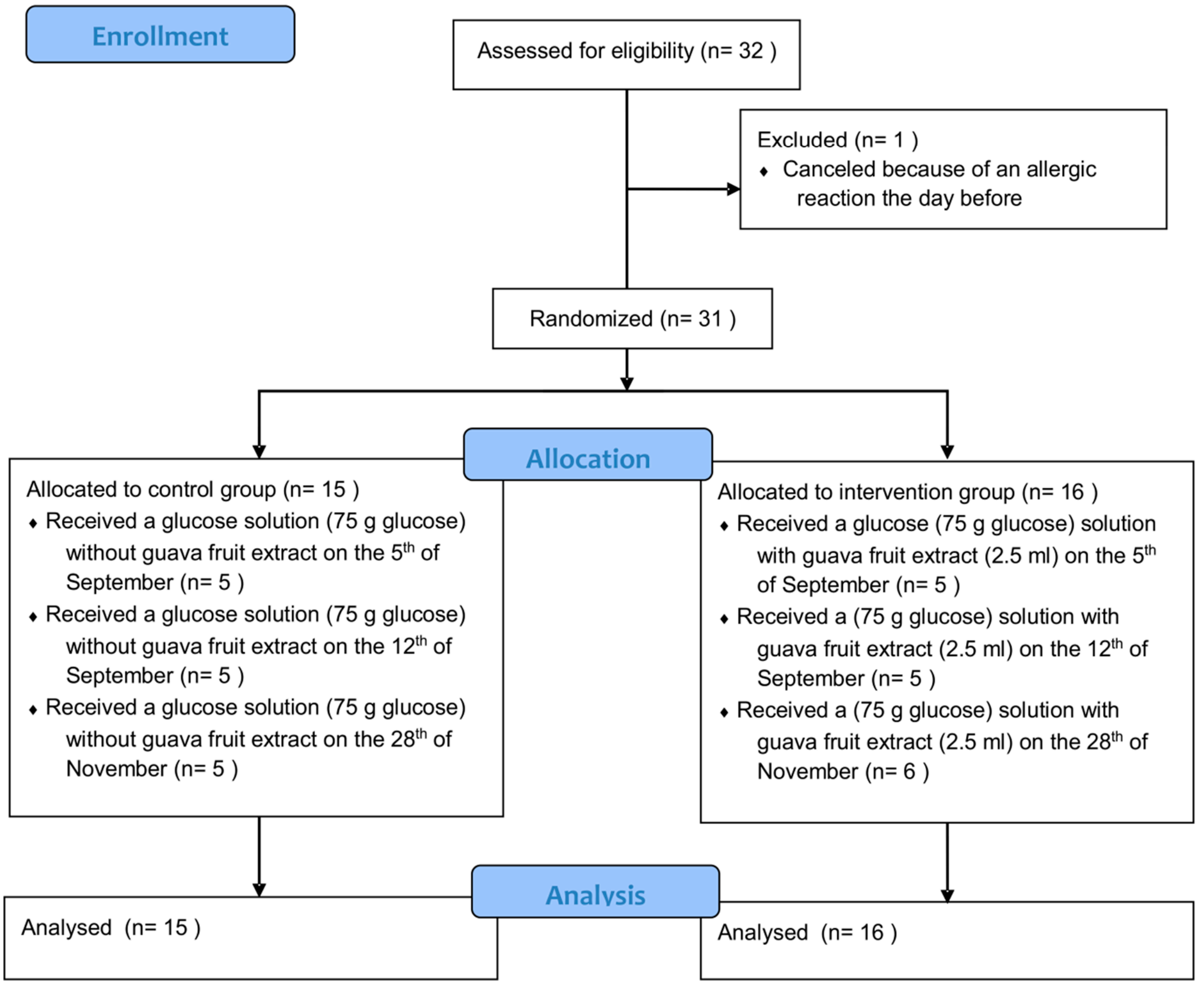

Supplementary Figure S1. Guava study CONSORT diagram.

Supplementary Figure 2

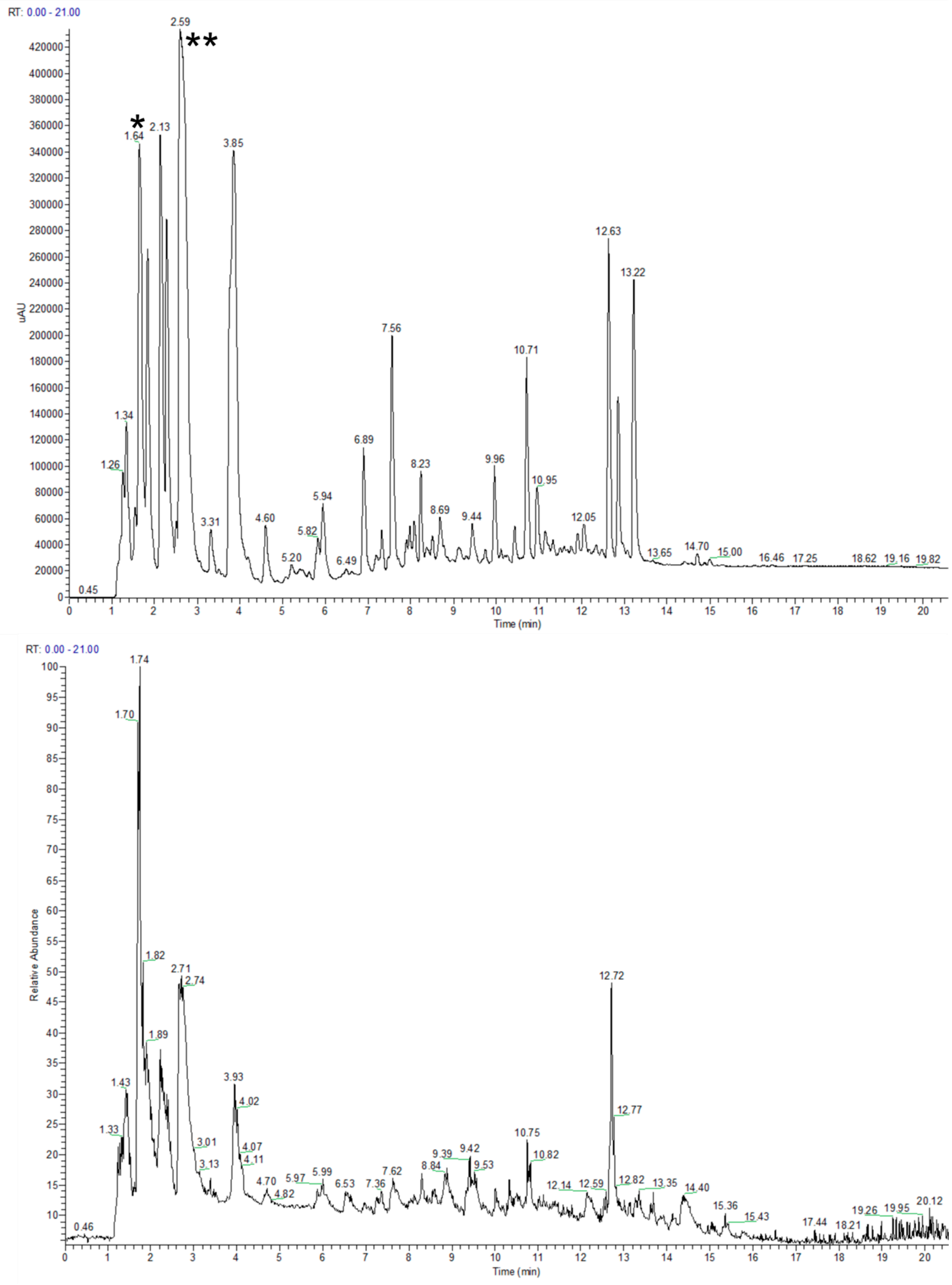

Supplementary Figure S2. HPLC-MS analysis of guava fruit extract prepared by supercritical CO_2 extraction. A diode array detector (DAD) chromatogram (top) and the total ion current (TIC) chromatogram (bottom) are shown. *Kojic acid (0.33 mg/mL, **5-Hydroxymethylfurfural (2.76 mg/mL).

Supplementary Figure 3

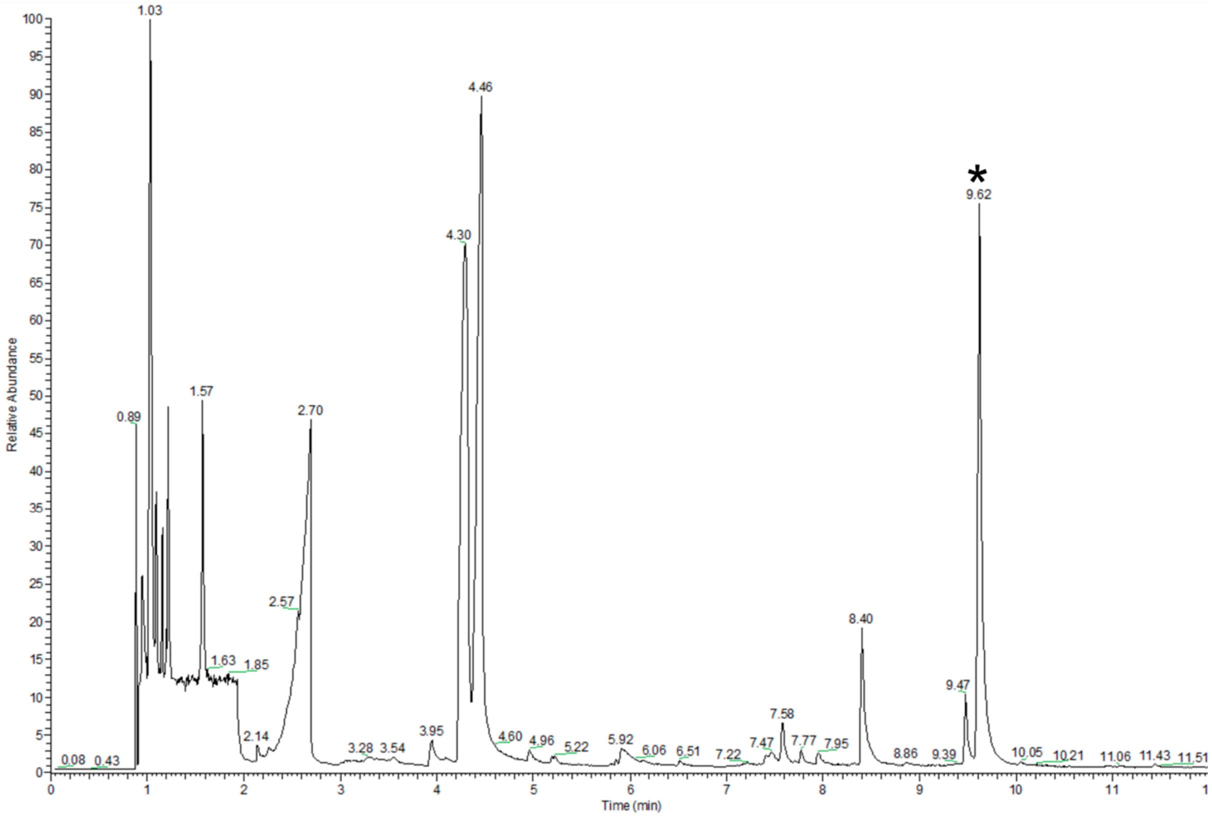

Supplementary Figure S3. GC-MS analysis of guava fruit extract prepared by supercritical CO_2 extraction. *5-Hydroxymethylfurfural.

Supplementary Figure 4

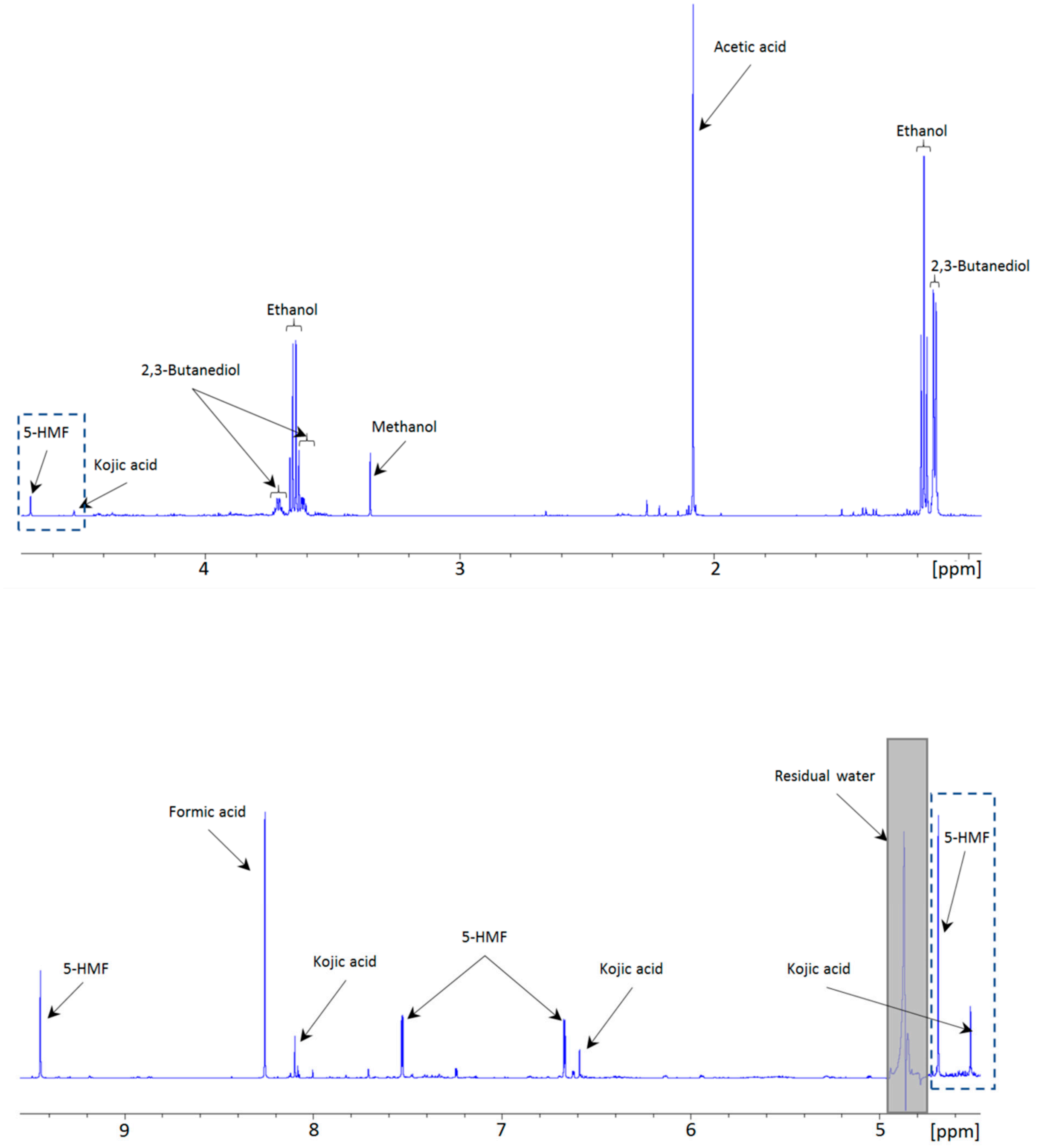

Supplementary Figure S4. NMR 1H-1D NMR spectrum of guava fruit extract prepared by supercritical CO_2 extraction. Upper panel shows the low range of the NMR-chemical shift scale (1.0 to 4.7 ppm) relevant for methanol, ethanol, acetic acid and 2,3-Butanediol. Lower panel depicts the region from 4.5 to 9.6 ppm relevant for 5-Hydroxymethylfurfural, Kojic acid, and Formic acid, respectively. The grey bar indicates residual water from water suppression. For better visibility, the signal intensities in both panels have been optimized (visible in the box with the broken line for signals belonging to 5-Hydroxymethylfurfural and Kojic acid).

.
